# 1,1,4,4-Tetra-*tert*-butyl-1,4-dichloro-2,2,3,3-tetra­phenyl­tetra­silane

**DOI:** 10.1107/S1600536812000669

**Published:** 2012-01-18

**Authors:** Kyohei Otsuka, Shintaro Ishida, Soichiro Kyushin

**Affiliations:** a Department of Chemistry and Chemical Biology, Graduate School of Engineering, Gunma University, Kiryu, Gunma 376-8515, Japan; b Department of Chemistry, Graduate School of Science, Tohoku University, Aoba-ku, Sendai 980-8578, Japan

## Abstract

The title compound, C_40_H_56_Cl_2_Si_4_, was synthesized by the coupling of 1,1-di-*tert*-butyl-1,2-dichloro-2,2-diphenyl­disilane with lithium. The asymmetric unit contains one half-mol­ecule, which is completed by an inversion centre. In the mol­ecule, the tetra­silane skeleton adopts a perfect *anti* conformation and the Si—Si bonds [2.4355 (5) and 2.4328 (7) Å] are longer than the standard Si—Si bond length (2.34 Å). The Si—Si—Si angle [116.09 (2)°] is larger than the tetra­hedral bond angle (109.5°). These long bond lengths and the wide angle are favorable for reducing the steric hindrance among the *tert*-butyl and the phenyl groups. The dihedral angle between the phenyl rings in the asymmetric unit is 37.36 (8)°.

## Related literature

For details of Wurtz-type reactions for formation of silicon–silicon bonds, see: Burkhard (1949 ▶); Gilman & Tomasi (1963 ▶); Stolberg (1963 ▶); Laguerre *et al.* (1978 ▶); Herman *et al.* (1985 ▶); Watanabe *et al.* (1988 ▶). For related structures of oligosilanes with *anti* conformations, see: Baumeister *et al.* (1997 ▶); Michl & West (2000 ▶); Tsuji *et al.* (2004 ▶); Fukazawa *et al.* (2006 ▶); Haga *et al.* (2008 ▶).
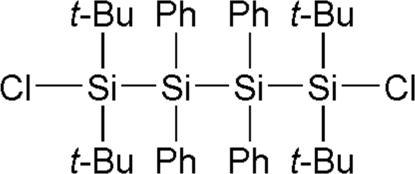


## Experimental

### 

#### Crystal data

C_40_H_56_Cl_2_Si_4_
*M* *_r_* = 720.11
Monoclinic, 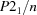

*a* = 9.6981 (8) Å
*b* = 15.3893 (11) Å
*c* = 13.8546 (11) Å
β = 105.7717 (7)°
*V* = 1989.9 (3) Å^3^
*Z* = 2
Mo *K*α radiation
μ = 0.31 mm^−1^
*T* = 153 K
0.30 × 0.10 × 0.10 mm

#### Data collection

Rigaku RAXIS-IV imaging plate diffractometer
Absorption correction: multi-scan (*REQAB*; Jacobson, 1998 ▶) *T*_min_ = 0.913, *T*_max_ = 0.970
12290 measured reflections
4895 independent reflections
4826 reflections with *I* > 2σ(*I*)
*R*_int_ = 0.020

#### Refinement

*R*[*F* ^2^ > 2σ(*F* ^2^)] = 0.037
*wR*(*F* ^2^) = 0.094
*S* = 1.10
4895 reflections
214 parameters
H-atom parameters constrained
Δρ_max_ = 0.32 e Å^−3^
Δρ_min_ = −0.32 e Å^−3^

### 

Data collection: *CrystalClear* (Rigaku, 2003 ▶); cell refinement: *CrystalClear*; data reduction: *CrystalClear*; program(s) used to solve structure: *SIR2004* (Burla *et al.*, 2005 ▶); program(s) used to refine structure: *SHELXL97* (Sheldrick, 2008 ▶); molecular graphics: *ORTEP-3* (Farrugia, 1997 ▶); software used to prepare material for publication: *SHELXL97* (Sheldrick, 2008 ▶) and *Yadokari-XG 2009* (Kabuto *et al.*, 2009 ▶).

## Supplementary Material

Crystal structure: contains datablock(s) global, I. DOI: 10.1107/S1600536812000669/is5036sup1.cif

Structure factors: contains datablock(s) I. DOI: 10.1107/S1600536812000669/is5036Isup2.hkl

Supplementary material file. DOI: 10.1107/S1600536812000669/is5036Isup3.cml

Additional supplementary materials:  crystallographic information; 3D view; checkCIF report

## Figures and Tables

**Fig. 1.**
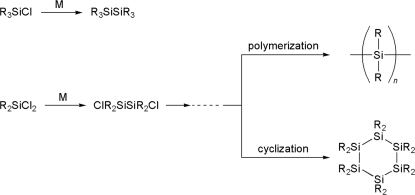
Wurtz-type reactions of trialkylchlorosilane and dialkyldichlorosilane.

**Fig. 2.**
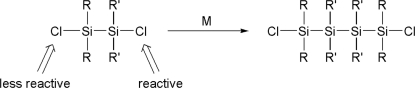
Synthesis of 1,4-dichlorotetrasilane by the Wurtz-type reaction of 1,2-dichlorodisilane.

**Fig. 3.**
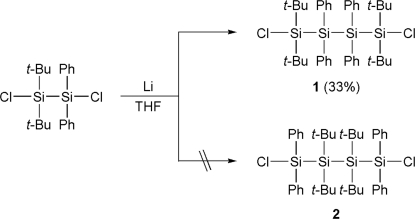
Synthesis of 1.

**Fig. 4.**
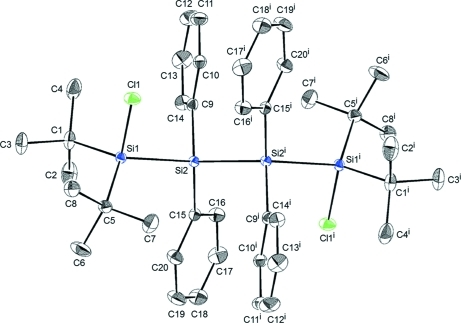
The molecular structure of the title compound, showing 50% probability displacement ellipsoids. [Symmetry code: (i) –x + 1, –y, –z + 1.]

## supplementary crystallographic information

### Comment

Wurtz-type reactions are the most general method of forming silicon–silicon bonds. Monochlorinated silanes such as chlorotrimethylsilane give disilanes by Wurtz-type reactions. When chlorosilanes containing two chlorine atoms are subjected to the Wurtz-type reactions, the reactions proceed successively to give polymerization and/or cyclization products as shown in Fig. 1. For example, the reaction of dichlorodimethylsilane with sodium gives poly(dimethylsilylene) (Burkhard, 1949), while the reaction of dichlorodimethylsilane with lithium in the presence of a catalytic amount of triphenylsilyllithium (Gilman *et al.*, 1963) gives dodecamethylcyclohexasilane. Also, the reaction of dichlorodimethylsilane with sodium–potassium alloy (Stolberg, 1963) or lithium (Laguerre *et al.*, 1978) gives dodecamethylcyclohexasilane. Similarly, the Wurtz-type reactions of 1,2-dichlorodisilanes have been reported to give polysilanes or cyclic oligosilanes, depending on reaction conditions (Herman *et al.*, 1985; Watanabe *et al.*, 1988).

If the Wurtz-type reactions of 1,2-dichlorodisilanes could be stopped at the stage of dimerization, it would be a convenient synthetic method for 1,4-dichlorotetrasilanes. To realise this method, one chlorosilyl moiety of 1,2-dichlorodisilane must be reactive in the Wurtz-type reaction, and the other must be less reactive. In this case, the reactive chlorosilyl moieties are coupled preferentially to afford 1,4-dichlorotetrasilane which does not react further as shown in Fig. 2. We report herein the synthesis of 1,4-dichlorotetrasilane by using 1,1-di-*tert*-butyl-1,2-dichloro-2,2-diphenyldisilane. In this compound, the chlorodiphenylsilyl moiety is expected to be more reactive than the di-*tert*-butylchlorosilyl moiety. We also report the X-ray crystal analysis of the resulting 1,4-dichlorotetrasilane.

The reaction of 1,1-di-*tert*-butyl-1,2-dichloro-2,2-diphenyldisilane with lithium (1.6 eq.) in tetrahydrofuran (THF) gave 1,1,4,4-tetra-*tert*-butyl-1,4-dichloro-2,2,3,3-tetraphenyltetrasilane 1 in 33% yield (Fig. 3). In this reaction, 2,2,3,3-tetra-*tert*-butyl-1,4-dichloro-1,1,4,4-tetraphenyltetrasilane 2 was not formed, indicating that the more reactive chlorodiphenylsilyl moieties are coupled predominantly. Unfortunately, the structure of 1 could not be determined by spectral data because compounds 1 and 2 are expected to show similar spectra. To distinguish these structures, we carried out the X-ray crystal analysis of 1.

The molecular structure of 1 is shown in Fig. 4. The tetrasilane skeleton of 1 adopts a perfect *anti* structure with an Si1—Si2—Si2^i^—Si1^i^ [symmetry code: (i) –*x* + 1, –*y*, –*z* + 1] torsion angle of 180.0° (Michl & West, 2000). The perfect or nearly perfect *anti* structures have rarely been reported in a few oligosilanes (Baumeister *et al.*, 1997; Tsuji *et al.*, 2004; Fukazawa *et al.*, 2006; Haga *et al.*, 2008). The silicon–silicon bonds [2.4355 (5) and 2.4328 (7) Å] are longer than the standard silicon–silicon bond (2.34 Å). The Si1—Si2—Si2^i^ bond angle [116.09 (2)°] is larger than the tetrahedral bond angle (109.5°). The long silicon–silicon bonds and the wide silicon–silicon–silicon bond angle are favorable for reducing the steric hindrance among the *tert*-butyl and phenyl groups. Four phenyl groups have almost perpendicular orientation to the tetrasilane plane to avoid the steric hindrance with the terminal *tert*-butyl groups.

### Experimental

A mixture of 1,1-di-*tert*-butyl-1,2-dichloro-2,2-diphenyldisilane (0.248 g, 0.627 mmol) and lithium (6.8 mg, 0.98 mmol) in THF (3 ml) was stirred at room temperature for 1 day. Ethanol (*ca.* 1 ml) was added to the reaction mixture, and the solvent was removed under reduced pressure. The residue was dissolved in chloroform and passed through a short column of silica gel. The filtrate was evaporated, and the residue was separated by column chromatography (silica gel, hexane–chloroform (1:1)) to give 1 (0.075 g, 33%) as colorless crystals. Single crystals were obtained from ethanol–THF (*ca.* 1:1) by slow evaporation.

1. Mp: 244–247 °C. ^1^H NMR (CDCl_3_): *δ* 1.01 (s, 36H), 7.45 (t, 8H, *J* = 7.6 Hz), 7.55 (t, 4H, *J* = 7.6 Hz), 7.94 (d, 8H, *J* = 7.6 Hz). ^13^C NMR (CDCl_3_): *δ* 26.1, 29.5, 127.4, 129.1, 134.0, 138.5. ^29^Si NMR (CDCl_3_): *δ* –32.0, 35.3.

### Refinement

All hydrogen atoms were generated at calculated positions and refined as riding atoms, with C—H = 0.95 (phenyl) or 0.98 (methyl) Å and *U*_iso_(H) = 1.2*U*_eq_(phenyl C) or 1.5*U*_eq_(methyl C).

### Crystal data

**Table:**

C_40_H_56_Cl_2_Si_4_	*F*(000) = 772
*M**_r_* = 720.11	*D*_x_ = 1.202 Mg m^−^^3^
Monoclinic, *P*2_1_/*n*	Melting point = 517–520 K
Hall symbol: -P 2yn	Mo *K*α radiation, λ = 0.71073 Å
*a* = 9.6981 (8) Å	Cell parameters from 9597 reflections
*b* = 15.3893 (11) Å	θ = 1.3–28.3°
*c* = 13.8546 (11) Å	µ = 0.31 mm^−^^1^
β = 105.7717 (7)°	*T* = 153 K
*V* = 1989.9 (3) Å^3^	Prism, colourless
*Z* = 2	0.30 × 0.10 × 0.10 mm

### Data collection

**Table:**

Rigaku RAXIS-IV imaging plate diffractometer	4895 independent reflections
Radiation source: rotating anode	4826 reflections with *I* > 2σ(*I*)
graphite	*R*_int_ = 0.020
Detector resolution: 10.00 pixels mm^-1^	θ_max_ = 28.3°, θ_min_ = 2.0°
ω scans	*h* = −12→12
Absorption correction: multi-scan (*REQAB*; Jacobson, 1998)	*k* = −20→20
*T*_min_ = 0.913, *T*_max_ = 0.970	*l* = −18→18
12290 measured reflections	

### Refinement

**Table:**

Refinement on *F*^2^	Primary atom site location: structure-invariant direct methods
Least-squares matrix: full	Secondary atom site location: difference Fourier map
*R*[*F*^2^ > 2σ(*F*^2^)] = 0.037	Hydrogen site location: inferred from neighbouring sites
*wR*(*F*^2^) = 0.094	H-atom parameters constrained
*S* = 1.10	*w* = 1/[σ^2^(*F*_o_^2^) + (0.0373*P*)^2^ + 1.7041*P*] where *P* = (*F*_o_^2^ + 2*F*_c_^2^)/3
4895 reflections	(Δ/σ)_max_ < 0.001
214 parameters	Δρ_max_ = 0.32 e Å^−^^3^
0 restraints	Δρ_min_ = −0.32 e Å^−^^3^

### Special details

**Table:**

Experimental. IR (KBr): 3080, 3050, 2980, 2950, 2940, 2890, 2850, 1470, 1430, 1390, 1370, 1360, 1180, 1090, 1010, 810, 730, 700 cm^–1^. MS (EI, 70 eV): *m*/*z* 541 (*M*^+^–177, 100), 359 (24), 324 (69), 267 (31), 259 (42), 197 (50), 183 (26), 135 (46).
Geometry. All e.s.d.'s (except the e.s.d. in the dihedral angle between two l.s. planes) are estimated using the full covariance matrix. The cell e.s.d.'s are taken into account individually in the estimation of e.s.d.'s in distances, angles and torsion angles; correlations between e.s.d.'s in cell parameters are only used when they are defined by crystal symmetry. An approximate (isotropic) treatment of cell e.s.d.'s is used for estimating e.s.d.'s involving l.s. planes.
Refinement. Refinement of *F*^2^ against ALL reflections. The weighted *R*-factor *wR* and goodness of fit *S* are based on *F*^2^, conventional *R*-factors *R* are based on *F*, with *F* set to zero for negative *F*^2^. The threshold expression of *F*^2^ > 2σ(*F*^2^) is used only for calculating *R*-factors(gt) *etc.* and is not relevant to the choice of reflections for refinement. *R*-factors based on *F*^2^ are statistically about twice as large as those based on *F*, and *R*-factors based on ALL data will be even larger.

### Fractional atomic coordinates and isotropic or equivalent isotropic displacement parameters (Å^2^)

**Table:**

	*x*	*y*	*z*	*U*_iso_*/*U*_eq_	
Si1	0.31115 (4)	0.00675 (2)	0.27448 (3)	0.01114 (9)	
Si2	0.52023 (4)	−0.00985 (2)	0.41781 (3)	0.00975 (9)	
Cl1	0.23186 (4)	0.12785 (2)	0.30352 (3)	0.01879 (9)	
C1	0.37002 (17)	0.02227 (11)	0.15322 (11)	0.0199 (3)	
C2	0.4726 (2)	−0.05206 (14)	0.14543 (13)	0.0315 (4)	
H1	0.4230	−0.1078	0.1431	0.047*	
H2	0.5563	−0.0508	0.2040	0.047*	
H3	0.5036	−0.0449	0.0843	0.047*	
C3	0.24115 (19)	0.02135 (13)	0.05901 (12)	0.0274 (4)	
H4	0.2749	0.0307	−0.0007	0.041*	
H5	0.1742	0.0677	0.0640	0.041*	
H6	0.1924	−0.0349	0.0539	0.041*	
C4	0.4457 (2)	0.11019 (13)	0.15219 (13)	0.0296 (4)	
H7	0.5316	0.1129	0.2091	0.044*	
H8	0.3805	0.1575	0.1574	0.044*	
H9	0.4731	0.1160	0.0894	0.044*	
C5	0.14973 (15)	−0.07002 (9)	0.25896 (11)	0.0165 (3)	
C6	0.16775 (19)	−0.15608 (11)	0.20623 (15)	0.0306 (4)	
H10	0.2618	−0.1812	0.2386	0.046*	
H11	0.1604	−0.1447	0.1354	0.046*	
H12	0.0924	−0.1969	0.2113	0.046*	
C7	0.13114 (18)	−0.09028 (13)	0.36307 (13)	0.0288 (4)	
H13	0.0449	−0.1256	0.3559	0.043*	
H14	0.1217	−0.0358	0.3974	0.043*	
H15	0.2150	−0.1223	0.4024	0.043*	
C8	0.00900 (16)	−0.02761 (11)	0.19730 (12)	0.0216 (3)	
H16	−0.0712	−0.0673	0.1939	0.032*	
H17	0.0158	−0.0153	0.1293	0.032*	
H18	−0.0069	0.0267	0.2296	0.032*	
C9	0.63923 (14)	0.08247 (9)	0.39841 (10)	0.0129 (3)	
C10	0.60184 (15)	0.16802 (9)	0.41542 (11)	0.0154 (3)	
H19	0.5174	0.1780	0.4360	0.018*	
C11	0.68493 (17)	0.23859 (10)	0.40297 (13)	0.0224 (3)	
H20	0.6580	0.2958	0.4161	0.027*	
C12	0.80733 (18)	0.22523 (11)	0.37132 (14)	0.0283 (4)	
H21	0.8644	0.2732	0.3625	0.034*	
C13	0.84559 (18)	0.14169 (12)	0.35275 (14)	0.0269 (4)	
H22	0.9293	0.1325	0.3310	0.032*	
C14	0.76306 (16)	0.07092 (10)	0.36547 (11)	0.0183 (3)	
H23	0.7907	0.0140	0.3517	0.022*	
C15	0.61049 (15)	−0.11866 (9)	0.41486 (10)	0.0125 (3)	
C16	0.75912 (16)	−0.13158 (10)	0.44964 (12)	0.0189 (3)	
H24	0.8197	−0.0833	0.4739	0.023*	
C17	0.81957 (17)	−0.21366 (11)	0.44931 (13)	0.0242 (3)	
H25	0.9206	−0.2204	0.4717	0.029*	
C18	0.73361 (19)	−0.28541 (11)	0.41658 (13)	0.0247 (3)	
H26	0.7753	−0.3413	0.4168	0.030*	
C19	0.58640 (19)	−0.27512 (11)	0.38349 (13)	0.0255 (3)	
H27	0.5264	−0.3241	0.3617	0.031*	
C20	0.52687 (16)	−0.19285 (10)	0.38224 (12)	0.0195 (3)	
H28	0.4258	−0.1866	0.3584	0.023*	

### Atomic displacement parameters (Å^2^)

**Table:**

	*U*^11^	*U*^22^	*U*^33^	*U*^12^	*U*^13^	*U*^23^
Si1	0.00980 (17)	0.01148 (18)	0.01079 (17)	0.00034 (13)	0.00048 (13)	−0.00035 (13)
Si2	0.00847 (17)	0.00997 (17)	0.01055 (17)	0.00025 (12)	0.00215 (13)	−0.00022 (12)
Cl1	0.01666 (17)	0.01261 (16)	0.02527 (19)	0.00270 (12)	0.00259 (13)	−0.00031 (12)
C1	0.0185 (7)	0.0299 (8)	0.0111 (6)	0.0007 (6)	0.0036 (5)	0.0007 (6)
C2	0.0296 (9)	0.0477 (11)	0.0194 (8)	0.0128 (8)	0.0104 (7)	−0.0017 (7)
C3	0.0251 (8)	0.0420 (10)	0.0124 (7)	−0.0017 (7)	0.0005 (6)	0.0021 (7)
C4	0.0291 (9)	0.0424 (10)	0.0174 (7)	−0.0104 (8)	0.0064 (6)	0.0062 (7)
C5	0.0122 (6)	0.0142 (6)	0.0187 (7)	−0.0024 (5)	−0.0033 (5)	0.0010 (5)
C6	0.0233 (8)	0.0164 (7)	0.0421 (10)	−0.0018 (6)	−0.0079 (7)	−0.0074 (7)
C7	0.0211 (8)	0.0375 (10)	0.0238 (8)	−0.0132 (7)	−0.0011 (6)	0.0097 (7)
C8	0.0134 (7)	0.0223 (7)	0.0246 (8)	−0.0004 (6)	−0.0024 (6)	0.0012 (6)
C9	0.0115 (6)	0.0152 (6)	0.0111 (6)	−0.0019 (5)	0.0015 (5)	0.0014 (5)
C10	0.0136 (6)	0.0152 (6)	0.0158 (6)	0.0001 (5)	0.0015 (5)	0.0011 (5)
C11	0.0200 (7)	0.0154 (7)	0.0276 (8)	−0.0031 (6)	−0.0007 (6)	0.0031 (6)
C12	0.0208 (8)	0.0245 (8)	0.0385 (10)	−0.0106 (6)	0.0064 (7)	0.0077 (7)
C13	0.0175 (7)	0.0319 (9)	0.0344 (9)	−0.0043 (7)	0.0123 (7)	0.0045 (7)
C14	0.0159 (6)	0.0198 (7)	0.0210 (7)	−0.0004 (5)	0.0081 (5)	0.0007 (5)
C15	0.0131 (6)	0.0131 (6)	0.0116 (6)	0.0027 (5)	0.0037 (5)	0.0003 (5)
C16	0.0146 (7)	0.0167 (7)	0.0242 (7)	0.0011 (5)	0.0033 (5)	0.0001 (6)
C17	0.0153 (7)	0.0240 (8)	0.0328 (9)	0.0089 (6)	0.0057 (6)	0.0044 (6)
C18	0.0277 (8)	0.0176 (7)	0.0298 (8)	0.0100 (6)	0.0095 (7)	−0.0009 (6)
C19	0.0255 (8)	0.0161 (7)	0.0318 (9)	−0.0001 (6)	0.0027 (7)	−0.0076 (6)
C20	0.0150 (6)	0.0172 (7)	0.0235 (7)	0.0018 (5)	0.0001 (5)	−0.0041 (6)

### Geometric parameters (Å, °)

**Table:**

Si1—C5	1.9261 (15)	C7—H15	0.9800
Si1—C1	1.9306 (15)	C8—H16	0.9800
Si1—Cl1	2.0963 (5)	C8—H17	0.9800
Si1—Si2	2.4355 (5)	C8—H18	0.9800
Si2—C15	1.8951 (14)	C9—C10	1.402 (2)
Si2—C9	1.8950 (14)	C9—C14	1.4072 (19)
Si2—Si2^i^	2.4328 (7)	C10—C11	1.391 (2)
C1—C3	1.542 (2)	C10—H19	0.9500
C1—C2	1.538 (2)	C11—C12	1.388 (2)
C1—C4	1.541 (2)	C11—H20	0.9500
C2—H1	0.9800	C12—C13	1.381 (3)
C2—H2	0.9800	C12—H21	0.9500
C2—H3	0.9800	C13—C14	1.391 (2)
C3—H4	0.9800	C13—H22	0.9500
C3—H5	0.9800	C14—H23	0.9500
C3—H6	0.9800	C15—C20	1.402 (2)
C4—H7	0.9800	C15—C16	1.4043 (19)
C4—H8	0.9800	C16—C17	1.393 (2)
C4—H9	0.9800	C16—H24	0.9500
C5—C7	1.533 (2)	C17—C18	1.384 (2)
C5—C6	1.545 (2)	C17—H25	0.9500
C5—C8	1.544 (2)	C18—C19	1.385 (2)
C6—H10	0.9800	C18—H26	0.9500
C6—H11	0.9800	C19—C20	1.390 (2)
C6—H12	0.9800	C19—H27	0.9500
C7—H13	0.9800	C20—H28	0.9500
C7—H14	0.9800		
			
C5—Si1—C1	113.73 (7)	C5—C7—H13	109.5
C5—Si1—Cl1	103.73 (5)	C5—C7—H14	109.5
C1—Si1—Cl1	105.53 (5)	H13—C7—H14	109.5
C5—Si1—Si2	119.87 (5)	C5—C7—H15	109.5
C1—Si1—Si2	110.17 (5)	H13—C7—H15	109.5
Cl1—Si1—Si2	101.79 (2)	H14—C7—H15	109.5
C15—Si2—C9	110.90 (6)	C5—C8—H16	109.5
C15—Si2—Si2^i^	108.86 (5)	C5—C8—H17	109.5
C9—Si2—Si2^i^	107.38 (5)	H16—C8—H17	109.5
C15—Si2—Si1	111.23 (5)	C5—C8—H18	109.5
C9—Si2—Si1	102.12 (4)	H16—C8—H18	109.5
Si2^i^—Si2—Si1	116.09 (2)	H17—C8—H18	109.5
C3—C1—C2	108.93 (14)	C10—C9—C14	117.10 (13)
C3—C1—C4	106.18 (14)	C10—C9—Si2	119.00 (10)
C2—C1—C4	109.48 (14)	C14—C9—Si2	123.89 (11)
C3—C1—Si1	111.84 (11)	C11—C10—C9	121.82 (14)
C2—C1—Si1	108.59 (11)	C11—C10—H19	119.1
C4—C1—Si1	111.77 (11)	C9—C10—H19	119.1
C1—C2—H1	109.5	C12—C11—C10	119.85 (15)
C1—C2—H2	109.5	C12—C11—H20	120.1
H1—C2—H2	109.5	C10—C11—H20	120.1
C1—C2—H3	109.5	C13—C12—C11	119.52 (15)
H1—C2—H3	109.5	C13—C12—H21	120.2
H2—C2—H3	109.5	C11—C12—H21	120.2
C1—C3—H4	109.5	C12—C13—C14	120.81 (15)
C1—C3—H5	109.5	C12—C13—H22	119.6
H4—C3—H5	109.5	C14—C13—H22	119.6
C1—C3—H6	109.5	C13—C14—C9	120.89 (15)
H4—C3—H6	109.5	C13—C14—H23	119.6
H5—C3—H6	109.5	C9—C14—H23	119.6
C1—C4—H7	109.5	C20—C15—C16	116.43 (13)
C1—C4—H8	109.5	C20—C15—Si2	119.76 (10)
H7—C4—H8	109.5	C16—C15—Si2	123.69 (11)
C1—C4—H9	109.5	C17—C16—C15	121.39 (14)
H7—C4—H9	109.5	C17—C16—H24	119.3
H8—C4—H9	109.5	C15—C16—H24	119.3
C7—C5—C6	109.13 (14)	C18—C17—C16	120.54 (14)
C7—C5—C8	107.10 (13)	C18—C17—H25	119.7
C6—C5—C8	107.30 (12)	C16—C17—H25	119.7
C7—C5—Si1	108.63 (10)	C19—C18—C17	119.50 (14)
C6—C5—Si1	112.68 (11)	C19—C18—H26	120.2
C8—C5—Si1	111.83 (10)	C17—C18—H26	120.2
C5—C6—H10	109.5	C18—C19—C20	119.71 (15)
C5—C6—H11	109.5	C18—C19—H27	120.1
H10—C6—H11	109.5	C20—C19—H27	120.1
C5—C6—H12	109.5	C19—C20—C15	122.41 (14)
H10—C6—H12	109.5	C19—C20—H28	118.8
H11—C6—H12	109.5	C15—C20—H28	118.8
			
C5—Si1—Si2—C15	63.66 (7)	Si2^i^—Si2—C9—C10	−50.23 (12)
C1—Si1—Si2—C15	−71.20 (7)	Si1—Si2—C9—C10	72.35 (11)
Cl1—Si1—Si2—C15	177.20 (5)	C15—Si2—C9—C14	12.38 (14)
C5—Si1—Si2—C9	−177.99 (7)	Si2^i^—Si2—C9—C14	131.21 (11)
C1—Si1—Si2—C9	47.15 (7)	Si1—Si2—C9—C14	−106.21 (12)
Cl1—Si1—Si2—C9	−64.44 (5)	C14—C9—C10—C11	−1.6 (2)
C5—Si1—Si2—Si2^i^	−61.54 (6)	Si2—C9—C10—C11	179.71 (12)
C1—Si1—Si2—Si2^i^	163.60 (6)	C9—C10—C11—C12	1.1 (2)
Cl1—Si1—Si2—Si2^i^	52.00 (3)	C10—C11—C12—C13	−0.2 (3)
C5—Si1—C1—C3	34.92 (14)	C11—C12—C13—C14	−0.1 (3)
Cl1—Si1—C1—C3	−78.12 (12)	C12—C13—C14—C9	−0.6 (3)
Si2—Si1—C1—C3	172.74 (11)	C10—C9—C14—C13	1.4 (2)
C5—Si1—C1—C2	−85.31 (13)	Si2—C9—C14—C13	179.96 (13)
Cl1—Si1—C1—C2	161.66 (11)	C9—Si2—C15—C20	−150.92 (11)
Si2—Si1—C1—C2	52.51 (12)	Si2^i^—Si2—C15—C20	91.15 (12)
C5—Si1—C1—C4	153.81 (11)	Si1—Si2—C15—C20	−38.00 (13)
Cl1—Si1—C1—C4	40.78 (12)	C9—Si2—C15—C16	33.11 (14)
Si2—Si1—C1—C4	−68.36 (12)	Si2^i^—Si2—C15—C16	−84.82 (12)
C1—Si1—C5—C7	169.63 (11)	Si1—Si2—C15—C16	146.04 (11)
Cl1—Si1—C5—C7	−76.26 (11)	C20—C15—C16—C17	1.4 (2)
Si2—Si1—C5—C7	36.25 (13)	Si2—C15—C16—C17	177.51 (12)
C1—Si1—C5—C6	48.58 (13)	C15—C16—C17—C18	−1.5 (3)
Cl1—Si1—C5—C6	162.69 (10)	C16—C17—C18—C19	0.3 (3)
Si2—Si1—C5—C6	−84.80 (12)	C17—C18—C19—C20	0.9 (3)
C1—Si1—C5—C8	−72.38 (12)	C18—C19—C20—C15	−0.9 (3)
Cl1—Si1—C5—C8	41.73 (11)	C16—C15—C20—C19	−0.2 (2)
Si2—Si1—C5—C8	154.24 (9)	Si2—C15—C20—C19	−176.46 (13)
C15—Si2—C9—C10	−169.06 (10)		

Symmetry codes: (i) −*x*+1, −*y*, −*z*+1.
